# Fine-Tuned Segment Anything Model with Low-Rank Adaptation for Chest X-Ray Images

**DOI:** 10.3390/diagnostics16060847

**Published:** 2026-03-12

**Authors:** Saeed S. Alahmari, Michael R. Gardner, Fawaz Alqahtani, Tawfiq Salem

**Affiliations:** 1 Department of Computer Science, Najran University, Najran 66462, Saudi Arabia; ssalahmari@nu.edu.sa; 2 Department of Biomedical Engineering, College of Engineering, King Faisal University, Al Ahsa 31982, Saudi Arabia; 3 Department of Radiological Sciences, College of Applied Medical Sciences, King Khalid University, Abha 61421, Saudi Arabia; 4 School of Applied and Creative Computing, Purdue University, West Lafayette, IN 47906, USA

**Keywords:** segmentation, SAM, chest X-ray, deep learning

## Abstract

**Background:** This paper investigates the use of the Segment Anything Model (SAM) for chest X-ray (CXR) image segmentation, with a focus on improving its performance using low-rank adaptation (LoRA). **Methods:** We evaluate three versions of SAM: two zero-shot methods (using coordinate and bounding box prompts) and a fine-tuned SAM using LoRA. To support these approaches, we also trained two standard convolutional neural networks (CNNs), U-Net and DeepLabv3+, to generate draft lung segmentations that serve as input prompts for the SAM methods. Our fine-tuning approach uses LoRA to add lightweight trainable adapters within the Transformer blocks of the SAM, allowing only a small subset of parameters to be updated. The rest of the SAM remains frozen, helping preserve its pre-trained knowledge while reducing memory and computational needs. We tested all models on a dataset of CXR images labeled for COVID-19, viral pneumonia, and normal cases. **Results:** Results show that fine-tuned SAM with LoRA outperforms zero-shot SAM methods and CNN baselines in terms of segmentation accuracy and efficiency. **Conclusions:** This demonstrates the potential of combining LoRA with SAM for practical and effective medical image segmentation.

## 1. Introduction

Chest X-rays (CXRs) are one of the most widely used diagnostic imaging modalities in clinical practice due to their low cost, speed, and effectiveness in detecting various thoracic diseases, including pneumonia and COVID-19 [1,2]. Accurate segmentation of anatomical regions, particularly the lungs, is a critical step in many automated CXR analysis systems. Segmentation enables downstream tasks such as disease localization, severity assessment, and treatment planning [3,4]. Traditional convolutional neural networks (CNNs), such as U-Net [5] and DeepLabv3+ [6], have demonstrated strong performance in medical image segmentation. However, these models often require large amounts of annotated data and extensive training time. Moreover, they are typically designed and trained for specific tasks, limiting their flexibility and generalizability to new domains or datasets [7,8]. The Segment Anything Model (SAM) [9], recently introduced by Meta AI, represents a new class of foundation models designed for general-purpose image segmentation. SAM is trained on a large and diverse dataset and can perform zero-shot segmentation using user-provided prompts such as points, bounding boxes, or masks. While promising, SAM’s performance in the medical domain—particularly on grayscale images like CXRs—remains largely unexplored. Early studies suggest that domain-specific fine-tuning is needed to adapt such large models for medical imaging tasks [10]. To address this, we explore the fine-tuning of SAM using low-rank adaptation (LoRA) [11], a parameter-efficient learning technique that enables targeted adaptation of pre-trained Transformer-based models. LoRA reduces the number of trainable parameters by injecting trainable low-rank matrices into existing model layers, allowing the base model to remain mostly frozen. This makes LoRA particularly attractive in medical imaging settings, where computational resources and labeled data are often limited. In this paper, we compare three SAM-based approaches for lung segmentation in CXR images: (1) zero-shot SAM with coordinate prompting, (2) zero-shot SAM with bounding box prompting, and (3) fine-tuned SAM using LoRA. For comparison, we also evaluate two CNN baselines (U-Net and DeepLabv3+) trained from scratch. We validate our methods on that include COVID-19, viral pneumonia, and normal CXR cases. Our contributions are summarized as follows:

1. We benchmark zero-shot SAM segmentation using coordinate and bounding box prompts derived from CNN-generated masks.
2. We propose a fine-tuning strategy for SAM using LoRA, tailored to the chest X-ray segmentation task.
3. We evaluate the performance of all models on curated CXR datasets, demonstrating that LoRA-tuned SAM outperforms both zero-shot methods and CNN baselines under constrained settings.

## 2. Background

Chest X-ray segmentation is essential for accurate diagnosis of diseases. Many approaches have been studied and developed for lung segmentation. An approach was proposed by Rajaraman et al. for the generalizability of lung segmentation from adult to pediatric cases. The authors performed a systematic evaluation and proposed the mean lung contour distance (MLCD) and average hash score (AHS) [12]. For tuberculosis chest X-ray segmentation and detection, a TB-UNet deep learning architecture was proposed by Iqbal et al. that uses dilated fusion blocks and attention blocks for lung segmentation. This work showed high precision and recall and was validated for different respiratory diseases such as pneumonia, COVID-19, and tuberculosis [13]. Since the original U-Net architecture is large in its number of parameters, a smaller and lighter-weight U-Net was proposed by Arvind et al. to avoid overfitting of the model [14]. This approach utilizes dropout in the deconvolution layers and was validated for semantic segmentation of chest X-rays using three datasets: the JSRT dataset, the Shenzhen dataset, and the MC dataset.

An attention-U-Net-based neural network that uses a general adversarial network [15] was proposed in [16] for lung segmentation. This approach was validated using the JSRT dataset and showed an average dice similarity coefficient of 97%. Another approach that uses an attention Transformer layer at the bottleneck was proposed by Din et al. [17]. This approach consists of four components: an encoder that is a pre-trained EfficientNet, a module for spatial enhancement in the skip connection, an attention Transformer module in the bottleneck layer, and a fusion of multi-scale features used for the decoder. This approach was validated using multiple chest X-ray datasets, including MC, Darwin, and Chenzhen datasets. Another study proposed Attention U-Net, where an attention module is added in the skipping connection of the U-Net layer for lung segmentation to enhance tuberculosis detection [18] using the Montgomery Chest X-ray dataset (https://www.kaggle.com/datasets/raddar/tuberculosis-chest-xrays-montgomery (accessed on 16 November 2025)) and Shenzhen Chest X-ray dataset (https://www.kaggle.com/datasets/raddar/tuberculosis-chest-xrays-shenzhen (accessed on 16 November 2025)). The authors also proposed a modified V-Net architecture, where they added a non-local block in the bottleneck layer. Furthermore, the authors modified and configured Seg-Net to use VGG16. The three neural network architectures were used for lung segmentation, where the proposed U-Net and V-Net showed the highest dice similarity coefficient compared to Seg-Net.

An experimental study by Hasan et al. uses DeepLabv3+ for the segmentation of the chest X-ray lung region [19]. This approach uses DeepLabv3+, which uses Atrous convolution and modified pooling layers. This approach has an average dice coefficient of 96.63% using the Chenzhen chest X-ray dataset. Another experimental study that uses an ensemble of four convolutional neural network models pre-trained on ImageNet as an encoder for the U-Net architecture was undertaken by Abedalla et al. [20]. The authors used four pre-trained models: ResNet50, DenseNet169, ResNext50, and EfficientNetB4. Furthermore, the authors experimented with stochastic weight averaging (SWA), test-time augmentation, and data augmentation. This experiment was done using the Pneumothorax challenge dataset (2019), where the ensemble neural network model showed an average dice similarity coefficient of 0.860. Similarly, a study was carried out by Rahman et al. for chest X-ray lung segmentation and tuberculosis detection [21]. The authors used a U-Net for lung segmentation and nine neural network pre-trained models for tuberculosis detection. The dataset for this approach was based on several public datasets, where the total chest X-ray images with tuberculosis is 3500, and the total number of normal chest X-ray images is 3500.

A modified U-Net architecture was proposed in [22]. This approach uses the EfficientNet-B4 model pre-trained on ImageNet as the encoder. Furthermore, a residual block and leakyReLU activation are used in the decoder. This modification showed an improvement in the Jaccard Index of 6% on two benchmark datasets, including the JSRT dataset [23] and Montgomery dataset [24]. Furthermore, this approach showed 9% improvement on a private chest X-ray dataset.

Precise lung segmentation improves the accurate detection of certain respiratory diseases. However, lung segmentation in chest X-ray images is challenging due to the acquisition settings, the disease’s impact on lung appearance, and the variation in lung shape. The authors of [25] applied multiple models, including vision Transformer models. The models are ARSeg [26], TransM [27], Medical Transformer [28], TransUNet [27], and UNeXt [29] for lung segmentation. The authors trained and validated these models using three datasets, including the Shenzhen chest X-ray dataset [30], the Montgomery chest X-ray dataset [24], and the JSRT chest X-ray dataset [23]. The best performing model achieved an average F1 score of 97.47%. Another study used the segmentation of chest X-ray lung region for the explainability of convolutional neural networks (CNNs) such as VGG16, ResNet, and Inception [31]. The authors applied the segmentation of the lung by masking out the non-lung region, then classified each image into pneumonia, COVID-19, or normal. The authors also assessed training on one source dataset and testing on the other source datasets. The findings indicate that there are biases inherited in the datasets that limit the generalizability of CNN models across datasets.

An approach was proposed to address the difficulty of segmenting the chest X-ray lung region due to the abnormality associated with opacities [32]. This approach patches the chest X-ray image, classifying each patch using a CNN model called AlexNet, then generates masks by constructing patches classified as lung, followed by post-processing. This approach was validated using the Montgomery chest X-ray dataset [24].

## 3. Dataset

To evaluate the proposed methodology, chest X-ray (CXR) images were sourced from a local hospital in the Najran region, Kingdom of Saudi Arabia. The image dataset comprises COVID-19 and normal chest X-ray images obtained from King Khalid Hospital (KKH) in Najran and viral pneumonia images collected from publicly available datasets [33]. Specifically, our dataset includes 552 COVID-19 CXR images acquired between June 2020 and May 2021. For normal images, 511 CXRs were gathered from KKH between March 2017 and February 2018 following a review of corresponding medical imaging reports. Viral pneumonia images, totaling 549, were sourced from publicly available datasets [33].

**Figure 1:**
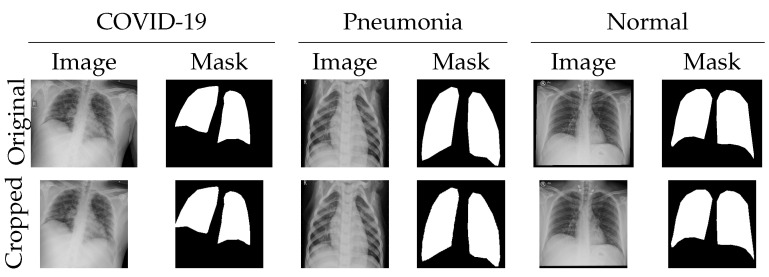
Images and masks from our dataset are resized and cropped to 224 × 224 pixel squares, avoiding any image distortion. The first column is for images and masks of cases labeled as COVID-19, the second column for images and masks of cases labeled as pneumonia, and the third column for images and masks of cases labeled as normal.

Ethical approval for this retrospective study was obtained from the institutional review boards of the General Directorate of Health Affairs, Najran (King Khalid Hospital). To enhance the dataset’s diversity, data augmentation techniques such as zooming and horizontal and vertical flipping were applied before model training. Table 1 summarizes our dataset, detailing the number of images per class and data sources. The dataset was split into training, validation, and testing subsets. The images in our datasets were resized and center-cropped to 244 × 244-pixel squares, avoiding any image distortion. Segmentation ground truth was collected by two researchers using the Label Studio tool. Example images from the dataset are shown in Figure 1.

## 4. Method

To conduct a comprehensive analysis of SAM networks for chest X-ray (CXR) segmentation, the SAM is examined through three distinct approaches: (1) a pre-trained zero-shot SAM utilizing coordinate prompts, (2) a pre-trained zero-shot SAM employing bounding box prompts, and (3) a fine-tuned SAM adapted using LoRA. The performance of these three SAM-based methods is further compared with two widely used convolutional neural networks (CNNs): (1) DeepLab-v3+ and (2) U-Net. These were both applied to CXR image segmentation. Since the zero-shot SAM approaches—based on coordinate and bounding box prompting—require an initial or “draft” segmentation of the image, this section first presents an overview of CNN-based segmentation before introducing and analyzing each of the three SAM-based methods.

### 4.1. Convolutional Neural Networks

We trained, validated, and tested two CNNs on an internal dataset of image/mask pairs partitioned into training (944), validation (329), and testing (311) sets. Both U-Net and DeepLabv3+ networks were trained from scratch using a stochastic gradient descent with momentum (SGDM) solver with an initial learn rate of 0.001. The maximum number of epochs was set to 5. Both networks were trained on a single-CPU MacBook Pro (2021) equipped with an Apple M1 Pro chip (Cupertino, CA, USA).

**Figure 2:**
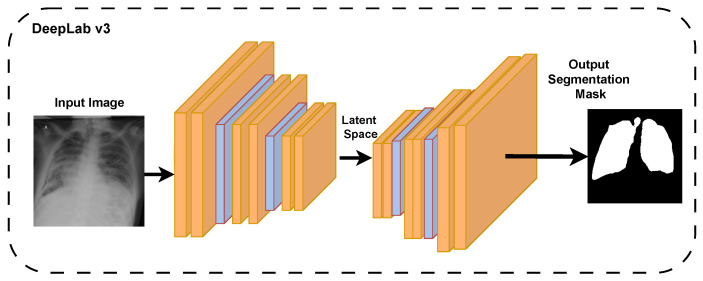
DeepLabv3 training for CXR images segmentation.

The two networks were assessed in terms of accuracy, intersection over union (IoU), and dice coefficient. The better-performing network was also used to segment images in the external dataset to test its generalizability. Its accuracy, dice coefficient, and IoU are also reported. In Figure 2, we show the DeepLabv3 training pipeline [34].

### 4.2. Zero-Shot SAM with Coordinate Prompting

To assess the zero-shot segmentation performance of the SAM on chest X-ray (CXR) images, the CNN model that demonstrated superior performance in our preliminary comparisons was selected as the base network for generating initial, or draft, lung segmentations. These preliminary segmentations were subsequently utilized to guide the SAM through two distinct prompting strategies: coordinate-based prompting and bounding-box-based prompting.

**Figure 3:**
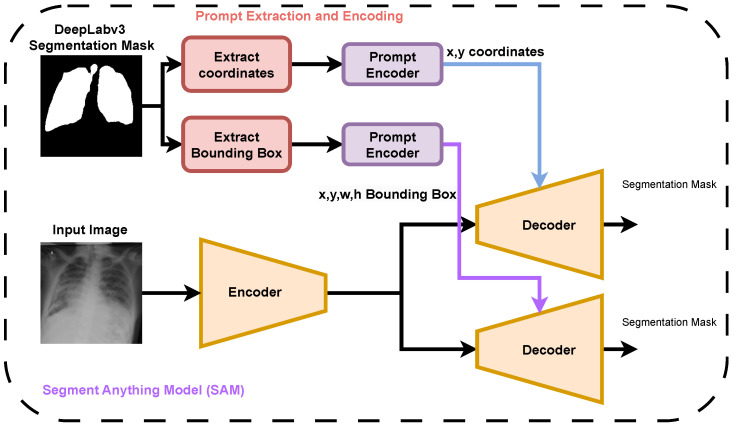
Zero-shot SAM where the input image is encoded, and the prompt is encoded for generating a segmentation mask.

In the coordinate-based prompting strategy for zero-shot SAM, a total of 105 prompting points were generated. These points were randomly sampled from the lung regions identified in the CNN-derived probability maps. The sampling process was probabilistically weighted such that pixels with higher lung-label probabilities had an increased likelihood of being selected as prompts. This approach ensured that the coordinate prompts were concentrated in high-confidence lung regions, thereby strengthening the reliability of the SAM’s zero-shot segmentation performance. The overall pipeline for generating both coordinate and bounding box prompts, based on DeepLabv3 segmentation masks, is illustrated in Figure 3, which demonstrates their role in guiding the SAM to produce segmentation masks for chest X-ray images.

### 4.3. Pre-Trained SAM with Bounding Box Prompting

For the bounding-box-based prompting strategy of the SAM, we utilized the lung segmentation masks generated by the CNN to derive tight bounding boxes around each lung in the chest X-ray images. These bounding boxes were computed by identifying the smallest rectangular region that fully encompassed the predicted lung area. Each derived bounding box, together with the corresponding raw chest X-ray image, was then provided as input to the SAM. Upon receiving this input, the SAM generated a segmentation output based on the spatial information provided by the bounding box, effectively leveraging the prior localization to guide its zero-shot segmentation predictions.

### 4.4. Fine-Tuned SAM Using LoRA

**Figure 4:**
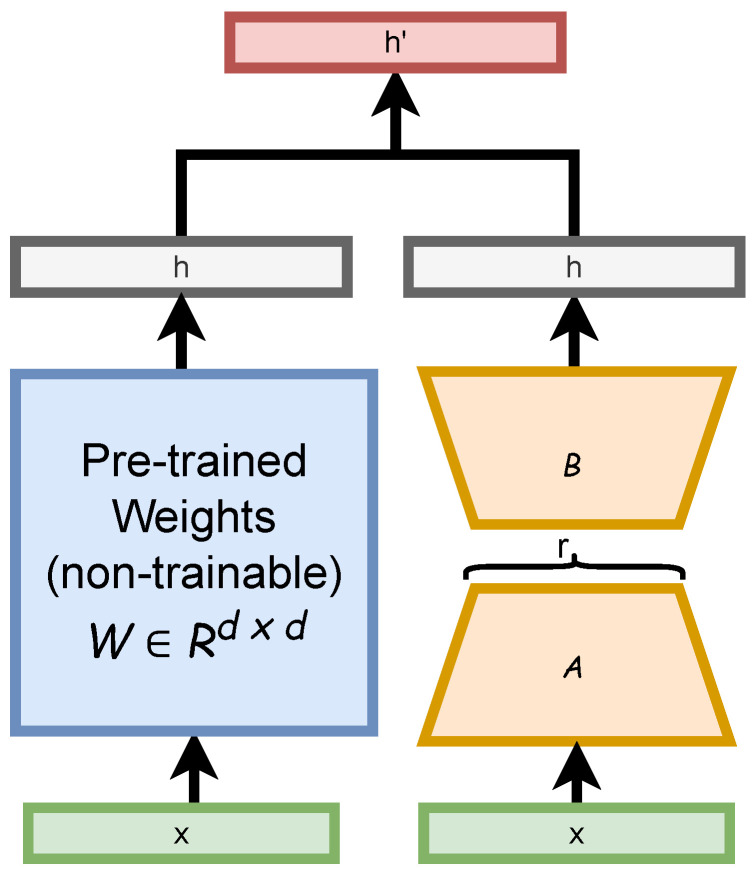
An illustration of LoRA approach for augmenting the original weights with A and B matrices.

In this section, we describe our approach to fine-tuning the SAM using the low-rank adaptation (LoRA) technique, which aims to reduce the number of trainable parameters while maintaining high performance. LoRA introduces low-rank matrix adapters into the Transformer architecture, enabling efficient training by adjusting only a small subset of weights. This method is particularly effective when computational resources or labeled data are limited. Figure 4 shows the design of the LoRA decomposition of the backpropagation matrix into two matrices with hyperparameter *r*.

**Figure 5:**
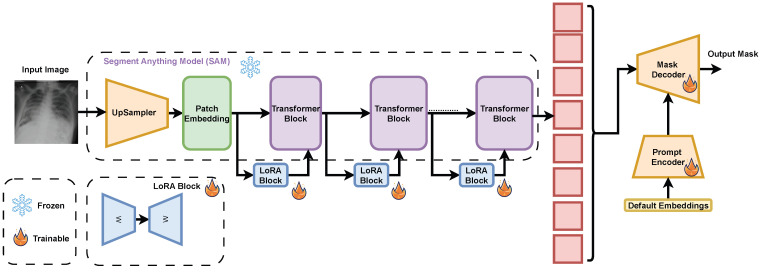
Fine-tuning SAM LoRA for segmentation of CXR images.

To accelerate the fine-tuning process of SAM, we integrated LoRA modules between each Transformer block in the image encoder, treating them as residual bypass connections. The original weights of the SAM were kept frozen to preserve the pre-trained knowledge, while only the newly introduced LoRA modules, the prompt encoder, and the mask decoder were set to be trainable. This selective training strategy enables targeted adaptation to the chest X-ray domain, significantly reducing memory and compute requirements compared to full model fine-tuning and making the approach more practical for medical imaging applications. The pipeline of fine-tuning is shown in Figure 5, where fine-tuning and testing of SAM were done on an NVIDIA GeForce 1080Ti GPU (Santa Clara, CA, USA).

## 5. Results and Discussion

In this section, we detail the results of our zero-shot assessment of the SAM versus other fine-tuning approaches, such as fine-tuning the SAM with LoRA. Table 2 presents the performance of the Segment Anything Model (SAM) in segmenting chest X-ray (CXR) images when varying the number of coordinate prompts with a zero-shot approach. The number of coordinates ranged from 15 to 105, increasing in steps of 15. Six evaluation metrics are reported: accuracy (%), intersection over union (IOU), dice coefficient, precision, recall, and F1-score. This experiment aimed to assess how increasing spatial supervision via coordinate prompts impacts segmentation quality in a zero-shot setting.

The results demonstrate a consistent performance improvement with more coordinate prompts. Starting with 15 points, the SAM achieved 56.2% accuracy, 0.409 IOU, 0.571 dice coefficient, 0.441 precision, 0.856 recall, and 0.571 F1-score. As the number of coordinates increased, all metrics showed steady gains. For example, at 60 coordinates, the accuracy rose to 59.6%, IOU to 0.439, dice coefficient to 0.599, precision to 0.463, recall to 0.891, and F1-score to 0.599.

The best overall performance was observed between 90 and 105 coordinates, where accuracy plateaued at approximately 60.3–60.6%, IOU reached up to 0.451, dice coefficient peaked at 0.611, precision peaked at 0.474, recall reached the highest value at 0.916, and F1-score reached the highest value at 0.611. These results suggest that the SAM benefits from a denser sampling of spatial cues, although the performance gains diminish beyond 75–90 coordinates, indicating a saturation point. This analysis reinforces the importance of prompt design in zero-shot SAM applications. It also provides a comparative baseline for evaluating the improvements achieved by our fine-tuned SAM using LoRA, which surpasses these results, as discussed in the subsequent sections.

In Table 3, we present a comprehensive comparison between three segmentation models—U-Net [5], DeepLabv3 [35], and SAM [9]—evaluated on the test set using six widely adopted performance metrics: average accuracy, average intersection over union (IoU), average dice coefficient, average precision, average recall, and average F1-score. These metrics were chosen to provide a balanced assessment of both pixel-level classification quality and region-level segmentation overlap. As shown in the table, fine-tuning the SAM on the newly curated dataset leads to a substantial and consistent improvement over all other evaluated configurations. Specifically, the fine-tuned SAM outperforms its zero-shot variant, as well as the U-Net and DeepLabv3 baselines, across all six metrics. This performance gain indicates that adapting the SAM to the target domain not only enhances its ability to accurately classify individual pixels but also improves its spatial consistency in capturing object boundaries and structural details. The superior IoU and dice coefficient scores further demonstrate the model’s effectiveness in reducing both false positives and false negatives in segmentation outputs. These results collectively suggest that while U-Net and DeepLabv3 remain competitive architectures in many segmentation tasks, the combination of the SAM’s powerful pre-trained representations with task-specific fine-tuning offers a more robust and generalizable approach for high-precision segmentation in this application domain.

**Figure 6:**
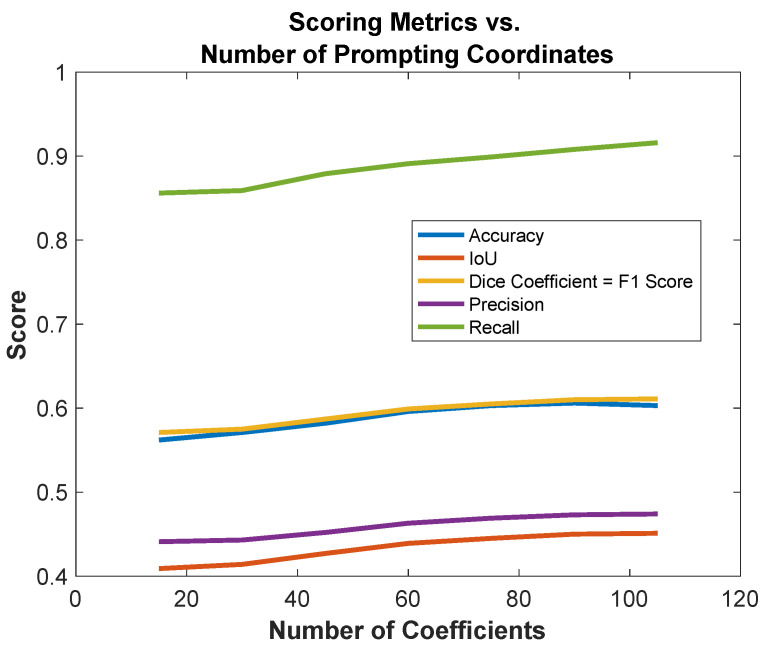
Comparison between the number of coordinates and accuracy, IoU, dice coefficient, precision, recall, and F1-score (**bottom**).

In Figure 6, we present the observed improvements in the zero-shot performance of the SAM when supplemented with additional spatial coordinates as part of the input prompt. The results indicate a slight upward trend in performance metrics—including accuracy, IoU, dice coefficient, precision, recall, and F1-score—as the number of spatial coordinates increases, demonstrating that providing the SAM with richer spatial context can slightly enhance its ability to delineate target regions without task-specific fine-tuning. This performance gain persisted up to the inclusion of 90 coordinates, beyond which a noticeable slight decline was observed for some metrics. The degradation in performance at higher coordinate counts suggests a potential saturation, where excessive prompting introduces redundant or noisy spatial information that may confuse the model rather than guide it effectively. Such findings highlight an important trade-off in prompt design: while additional spatial cues can be beneficial, over-selecting coordinates may overwhelm the model’s inference process, thereby impairing its segmentation capabilities under a zero-shot setting. This observation underscores the need for careful calibration of prompt complexity for better segmentation results.

**Figure 7:**
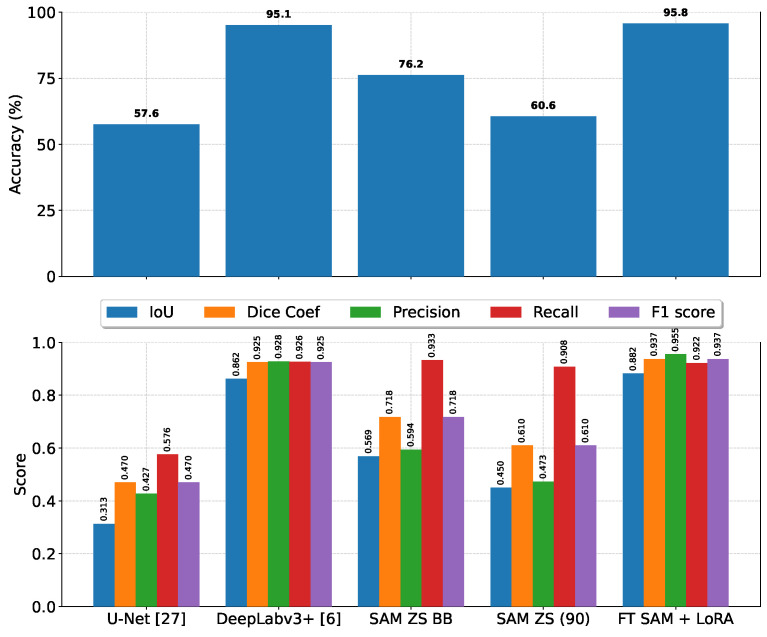
Avisual comparison between different segmentation methods for lungs in CXR images, where SAM ZS BB denotes the SAM zero-shot bounding box approach, SAM ZS (90) denotes the SAM zero-shot with 90 coordinates, and FT SAM + LoRA denotes fine-tuned SAM + LoRA [6,27].

In Figure 7, we provide a visualization for comparing U-Net, DeepLabv3+, SAM zero-shot coordinate-based prompting, SAM zero-shot bounding-box-based prompting, and fine-tuned SAM with LoRA capabilities, which shows the superiority of the fine-tuned SAM over all other models.

**Figure 8:**
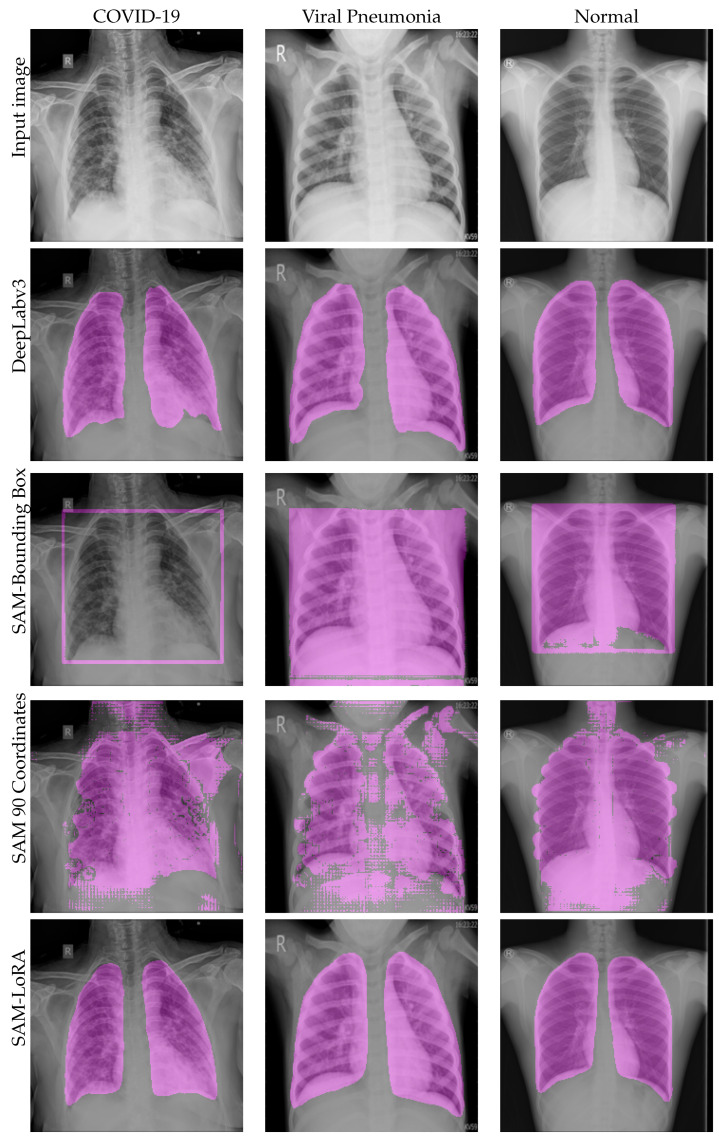
Avisualization of the performance of different models on three categories of chest X-ray images. The masks are overlaid on the original image in purple.

A comparative visualization between DeepLabv3, SAM zero-shot with coordinates (90 coordinates), SAM zero-shot with bounding box, and fine-tuned SAM with LoRA is provided in Figure 8. The SAM bounding box underperformed compared with the other approaches. DeepLabv3 performance and the fine-tuned SAM were mostly similar on the visualization.

## 6. Conclusions

This study investigated the use of the Segment Anything Model (SAM) for chest X-ray lung segmentation and introduced a lightweight fine-tuning strategy using low-rank adaptation (LoRA). We compared zero-shot SAM approaches with coordinate and bounding box prompts against CNN baselines (U-Net and DeepLabv3+), showing that while prompt design improves zero-shot results, fine-tuned SAM with LoRA achieved the best overall performance across accuracy, IoU, and dice coefficient metrics. These results demonstrate the value of adapting foundation models like SAM to medical imaging and highlight LoRA as an efficient method for achieving high-quality segmentation with limited computational resources. Future work will explore extending this approach to other imaging modalities and tasks, further reducing annotation needs and supporting scalable clinical applications.

## Figures and Tables

**Table 1:** Summary of image dataset and source.

Image Class	Number of Images	Source
COVID-19	552	KKH
Normal	511	KKH
Viral Pneumonia	549	Chowdhury [33]

**Table 2:** Performance of SAM using prompts with different numbers of coordinates, starting at 15 with an increment of 15 coordinates up to 105 coordinates.

Number of Coordinates	Accuracy %	IoU	Dice Coefficient	Precision	Recall	F1 Score
15	56.2	0.409	0.571	0.441	0.856	0.571
30	57.1	0.414	0.575	0.443	0.859	0.575
45	58.2	0.427	0.587	0.452	0.879	0.587
60	59.6	0.439	0.599	0.463	0.891	0.599
75	60.3	0.445	0.605	0.469	0.899	0.605
90	60.6	0.450	0.610	0.473	0.908	0.610
105	60.3	0.451	0.611	0.474	0.916	0.611

**Table 3:** Comparison between the segmentation performance of U-Net, DeepLabv3, and SAM.

Number of Coordinates	Accuracy %	IoU	Dice Coef	Precision	Recall	F1 Score
U-Net [5]	57.6	0.313	0.470	0.427	0.576	0.470
DeepLabv3+ [35]	95.1	0.862	0.925	0.928	0.926	0.925
SAM zero-shot bounding box	76.2	0.569	0.718	0.594	0.933	0.718
SAM zero-shot (90 coord)	60.6	0.450	0.610	0.473	0.908	0.610
Fine-tuned SAM + LoRA	95.8	0.882	0.937	0.955	0.922	0.937

## Data Availability

Viral pneumoniadata presented in the study are openly available as reported in [33]. COVID-19 and normal data are withheld for patient privacy but available upon request.

## Abbreviations

The following abbreviations are used in this manuscript:

CXR	Chest X-ray
LoRA	Low-rank adaptation
CNN	Convolutional neural network
SAM	Segment anything model
